# Efficacy of Acupoints Dual-Frequency Low-Level Laser Therapy on Knee Osteoarthritis

**DOI:** 10.1155/2020/6979105

**Published:** 2020-09-24

**Authors:** Fang-Yin Liao, Chien-Lin Lin, Sui-Foon Lo, Chun-Ching Chang, Wen-Yen Liao, Li-Wei Chou

**Affiliations:** ^1^Department of Physical Medicine and Rehabilitation, China Medical University Hospital, Taichung 40432, Taiwan; ^2^School of Chinese Medicine, College of Chinese Medicine, China Medical University, Taichung 40433, Taiwan; ^3^Department of Rehabilitation, New Bodhi Hospital, Taichung 41222, Taiwan; ^4^Department of Physical Therapy and Graduate Institute of Rehabilitation Science, China Medical University, Taichung 40433, Taiwan; ^5^Department of Physical Medicine and Rehabilitation, Asia University Hospital, Asia University, Taichung 41350, Taiwan

## Abstract

**Background:**

Knee osteoarthritis (OA) presented with knee pain and limitation of mobility is common, and it may become a chronic problem resulting in major loss of function, with related impaired activity of daily living. Current traditional therapy for knee OA includes pharmacological treatment and physiotherapy, but the efficacies are limited. An alternative noninvasive treatment low-level laser therapy (LLLT) applied to acupoints is still contradictory and the efficacy needs to be assessed.

**Methods and Materials:**

We conduct the randomized double-blind control study to investigate the efficacy of a dual-frequency LLLT (combines red light (780 nm) and near-infrared light (830 nm)) in patients suffering knee OA. Participates were randomly assigned into active laser therapy (ALT) and placebo laser therapy (PLT) groups. Subjects in the ALT group were separately treated by laser apparatus at the three acupoints (SP9, SP10, and EX-LE2) on their knee joints under continuous radiation for 15 min at the maximum intensity, three times per week for four weeks. The PLT group used laser apparatus of the same model according to similar procedures without laser light emission. Outcome Measurements including visual analog scale (VAS), pain pressure threshold (PPT), and Lequesne index were used.

**Results:**

A total of 30 subjects with two-sided knee OA in both groups completed the experiment. Statistically significant decreases were observed in the Lequesne index (5.27 ± 3.26 vs. 10.83 ± 3.83), conscious VAS 4 weeks after treatment (moving: 2.87 ± 1.13 vs. 5.67 ± 1.72; resting: 0.33 ± 0.62 vs. 2.67 ± 1.29), and the increase was noted in PPT (21.23 ± 1.82 kg vs. 13.02 ± 1.46 kg) in the ALT group compared with the PLT group.

**Conclusion:**

It appears that the knee OA pain and disability can be decreased after a dual-frequency LLLT applied to acupoints (SP9, SP10, and EX-LE2). The clinical efficacy of LLLT is highly related to the therapeutic settings of the laser apparatus; hence, more clinical trials with diffident parameter settings are needed to be further clarified.

## 1. Introduction

In the modern society, degenerative arthritis is one of the common diseases prevalent among the population over age 45; the incidence of this disease is twice more prominent in females than in male [1–7]. Damages to peripheral soft tissues, including the bursa, tendon, and ligament around the joint, are probable sources of pain in patients with degenerative arthritis, which is a joint deformity, caused by articular cartilage wear and tear. In imaging, knee osteoarthritis (OA) can be divided into four levels by severity. However, the results of X-ray examination are not exactly the same as the clinical manifestations [1, 8, 9]. Therefore, the American College of Rheumatology (ACR) formulated diagnostic criteria for knee OA: over age 50; early morning stiffness for less than 30 minutes; a sudden bursting sound as the ball joint moves; knee pain or tenderness; and joint space narrowing, spur proliferation, and bone hypertrophy as revealed by X-ray imaging [10].

Degenerative arthritis is manifested with joint pain and impaired physical mobility, belonging to the category of arthromyodynia in traditional Chinese medicine. According to Huangdi Neijing, although clinical manifestations of arthromyodynia vary with depth and location, the blocked or unbalanced Qi or blood circulation through the body's meridians can be relieved by acupuncture at certain points, mainly involving five-point acupuncture of knee, Yangming stomach channel of foot, Shaoyang gall bladder channel of foot, and Taiyin spleen channel of foot.

Traditional therapeutic options for degenerative arthritis mainly include medication and physiotherapy in rehabilitation department [11, 12]. However, the former is prone to side effects, whereas the latter makes little effects in some severe cases. Thus, low-level laser therapy (LLLT), a noninvasive substitutive therapy, is adopted in the present study [13–15]. Laser therapy in animal experiments can adjust the proliferation of chondrocytes and the synthesis of DNAs in cartilage-specific proteoglycan and collagen/noncollagen proteins (NCPs) [16–18], relieve pain, accelerate the release of serotonin from the body [19], and produce an anti-inflammatory effect [20]. In the present study, bichromatic laser-emitting dual-frequency laser beams in an alternating way can achieve deeper laser penetration, which may produce a better curative effect despite variation of lesion depth from person to person and from spot to spot [21]. Based on the literature, three acupoints, which are commonly used to treat knee OA, including SP9 (yīnlíngqúan, Taiyin spleen channel of foot), SP10 (xuěhǎi, Taiyin spleen meridian of foot), and EX-LE2 (hèdǐngxué) [22–24], were chosen in the present study. A double-blind trial was conducted to determine the curative effect of acupoints dual-frequency LLLT on knee osteoarthritis.

## 2. Materials and Methods

This study involved patients diagnosed with knee OA at rehabilitation clinics. This study was approved by the Institutional Review Board of the China Medical University Hospital (DMR99-IRB-136). Patients gave their written informed consent to participate in this study, and the research was conducted in accordance with the principles of the Declaration of Helsinki.

The inclusion criteria were (1) knee OA patients who meet the criteria established by the ACR on 1986; (2) those with knee OA as revealed by X-ray imaging, having the severity above Level II as rated by the Kellgren–Lawrence grading scale; (3) those over the age of 50; (4) those with knee OA pain lasting clinically for more than six months; and (5) those who are willing to cooperate and sign informed consents after being fully apprised of procedures and its purposes of the clinical trial. The exclusion conditions were (1) those suffering from malignant tumors and/or acute medical diseases, motor or sensory nerve defect, mental disorders, dementia, mental retardation, and/or any other organic mental abnormality; (2) those who have received intra-articular steroids or hyaluronic acid in their knees over the past three months; (3) those who have ever undergone knee surgeries, or whose knees have been wounded and suffer from congenital knee deformation, severe knee varus or valgus deformity, or secondary knee OA to endocrinopathy, metabolic disorders, infectious and inflammatory diseases, and/or rheumatic autoimmune diseases; and (4) those with contraindications for LLLT, such as malignant tumors, pregnancy, and photosensitization. After the screening, the eligible subjects were completely randomly assigned to the treatment group (*n* = 16) and the control group (*n* = 17) by means of random number table. The randomization procedure was performed by a researcher who was not involved in data collection, and assignments were held in sealed opaque envelopes to ensure randomization concealment. During the follow-up course, there were one or two patients walking out from the treatment group and the control group, respectively. Thus, the two groups included 15 subjects each at the completion of the course, which was conducted three times per week for four weeks.

**Figure 1:**
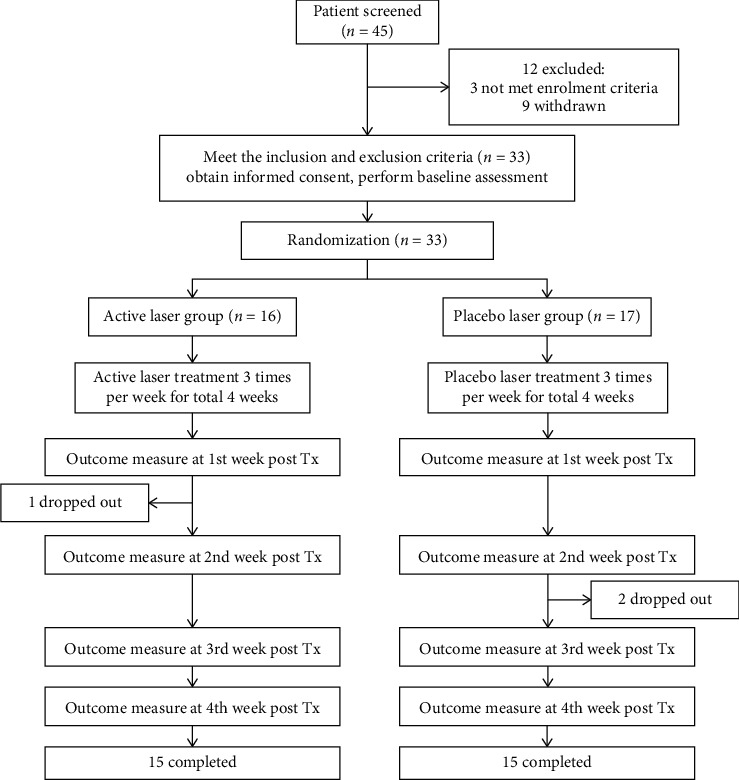
Flowchart of this study.

In the present study, multiband laser therapeutic apparatuses (TI-816-2, Transverse Inc., Taiwan) were adopted as the active laser treatment (ALT) group, with the output wavelengths of 780 nm (power: 50 mW) and 830 nm (power: 30 mW), and the maximum cumulative energy of 216 J under the continuous irradiation for 15 min at the maximum intensity. The clinical trial was designed as a randomized, double-blind, and controlled trial. Subjects were separately treated by laser apparatus at the three points (SP9, SP10, and EX-LE2) on their knee joints under continuous radiation for 15 min at the maximum intensity, three times per week for four weeks. The control group (placebo laser treatment (PLT)) in the experiment used laser apparatus of the same model according to similar procedures, but without laser light emission. The flowchart of this study is shown in Figure 1.

The efficacy was assessed by three outcome measurements: chief complaint about pain intensity (both moving and resting knee), pain pressure threshold (PPT), and knee OA severity scale. The chief complaint about pain intensity is expressed by the visual analog scale (VAS). This method is represented with a straight line of 10 cm, both ends of which are marked with “0” and “10” (“0”: completely painless; “10”: intolerable pain), which allows patients to mark and identify their pain levels by measuring the distance between the point of “0” and the corresponding markers, indicating their respective pain index values. Pressure algometry was applied to measure the PPT three times at a certain sore spot of each patient's pes anserinus tendon, with an interval of 60 seconds, and the value of pressure algometry at which pain became unbearable was recorded. Additionally, the median of the three measured values was defined as the pain pressure threshold. The Lequesne index [25] was measured by the knee osteoarthritis severity scale. By summing up, the score for the lowest severity level was 0 point, and that of the highest severity level was 24 points.

The results from the present study were statistically analyzed using Statistical Package for Social Science (SPSS 18.0) for Windows. Data are expressed as mean ± standard deviation (SD). As for descriptive statistics, the between-group analysis of all variables was conducted by independent two-sample *t*-test and chi-square test, thereby obtaining the curative effects. For inferential statistics, the within-group analysis of all variables was conducted by the paired sample *t*-test while the between-group analysis of the variables was conducted by the independent two-sample *t*-test. As revealed in the present study, there were statistically significant differences among the results (*P* < 0.05).

## 3. Results

In the present study, a total of 33 subjects were randomized into two groups (ALT and PLT). The ALT group consisted of 16 subjects while the PLT group was of 17 subjects. However, one or two subjects had withdrawn from the ALT and PLT groups, respectively, in the middle of the study period; thus, the two groups had 15 subjects at the completion of the therapy for four weeks (see Figure 1). A total of 30 patients with two-sided knee OA completed the study. After diagnosis, a total of 60 knee OA samples were collected. Statistically, the mean values of clinical index data of every subject's left and right knees were taken for clinical assessment. There were no statistically significant differences between the two groups in basic data (Table 1). In terms of clinical evaluation indicators before therapy, including knee OA severity index, conscious VAS for moving knee, conscious VAS for resting knee, and pes anserinus tendon PPT (Table 1), there were no statistically significant differences between the two groups in those clinical evaluation indicators before therapy (independent two-sample *t*-test).

### 3.1. Severity Index of Knee OA Severity (Lequesne Index)

**Figure 2:**
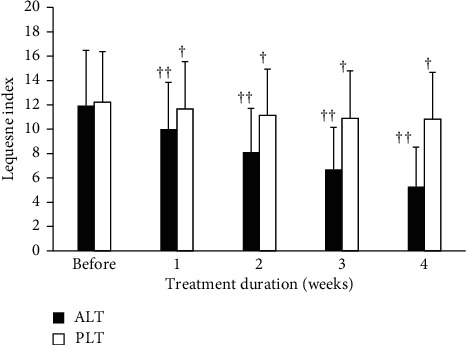
The variations of the two groups in the knee OA severity index before experiment and 4 weeks after treatment. Data are expressed as bar plot (mean) with error bars (SD) (^†^*P* < 0.001, ^††^*P* < 0.0001) (ALT: active laser treatment; PLT: placebo laser treatment).

The variations of the two groups in the knee OA severity index before experiment and within the 1st, 2nd, 3rd, and 4th weeks after treatment are shown as Figure 2. Comparison of the ALT group before LLLT and within the 1st, 2nd, 3rd, and 4th weeks after treatment showed that the knee OA severity index was significantly improved with statistically significant differences. By contrast, the Lequesne index of the PLT group after the four-week course did not significantly decrease, and the therapeutic regimen for the PLT group produced little curative effect on knee OA from the Lequesne index.

### 3.2. Conscious VAS for Moving Knee

**Figure 3:**
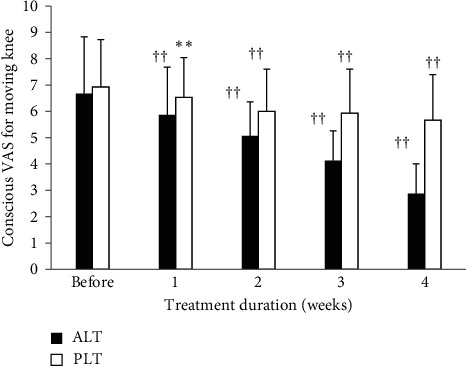
The variations of the two groups in the conscious VAS for moving knees before experiment and 4 weeks after treatment. Data are expressed as bar plot (mean) with error bars (SD) (^*∗∗*^*P* < 0.01, ^††^*P* < 0.0001) (ALT: active laser treatment; PLT: placebo laser treatment).

Considering the conscious VAS for moving knees, the results of the two groups before experiment and within the 1st, 2nd, 3rd, and 4th weeks after it are shown in Figure 3. Results showed that, for the ALT group before LLLT and within the 1st, 2nd, 3rd, and 4th weeks after it, the conscious VAS for moving knee significantly decreased (6.67 ± 2.16, before; 2.87 ± 1.13, within the 4th week after). However, the conscious VAS of the PLT group after the four-week therapy did not drop significantly (6.93 ± 1.79, before; 5.67 ± 1.72, within the 4th week after). Significant differences were found between the two groups in the experimental results.

### 3.3. Conscious VAS for Resting Knee

**Figure 4:**
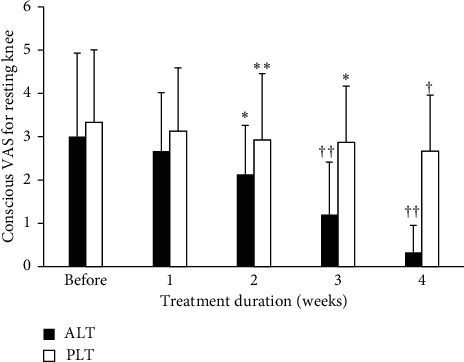
The variations of the two groups in the conscious VAS for resting knees before experiment and 4 weeks after treatment. Data are expressed as bar plot (mean) with error bars (SD) (^*∗*^*P* < 0.05, ^*∗∗*^*P* < 0.01, ^†^*P* < 0.001, ^††^*P* < 0.0001) (ALT: active laser treatment; PLT: placebo laser treatment).

The variations of the two groups in the conscious VAS for resting knee before experiment and within the 1st, 2nd, 3rd, and 4th weeks after it are shown in Figure 4. By comparing the ALT group before LLLT and within the 1st, 2nd, 3rd, and 4th weeks after it, the conscious VAS for resting knee was significantly improved (3.00 ± 1.93, before; 0.33 ± 0.62, within the 4th week after), having statistically significant differences. For the PLT group, the conscious VAS for resting knee was not significantly improved (3.33 ± 1.68, before; 2.67 ± 1.29, within the 4th week after).

### 3.4. Pes Anserinus Tendon Pain Pressure Threshold

**Figure 5:**
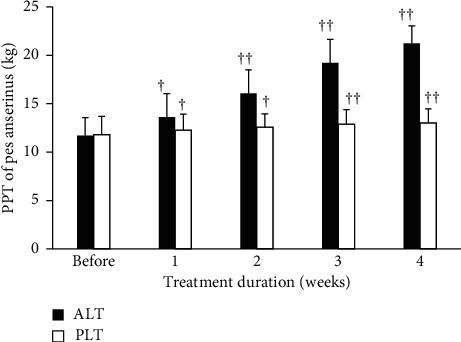
The variations of the two groups in the pes anserinus tendon pain pressure threshold before experiment and 4 weeks after treatment. Data are expressed as bar plot (mean) with error bars (SD) (^†^*P* < 0.001, ^††^*P* < 0.0001) (ALT: active laser treatment; PLT: placebo laser treatment).

The variations of the two groups before experiment and within the 1st, 2nd, 3rd, and 4th weeks after it in the pes anserinus tendon PPT are shown as Figure 5. Results showed that, for the ALT group before LLLT and within the 1st, 2nd, 3rd, and 4th weeks after it, the pes anserinus tendon PPT was significantly improved (11.71 ± 1.86, before; 21.23 ± 1.82, within the 4th week after), having statistically significant differences. The PPT of the PLT group was 12–13, indicating that the therapeutic regimen for the PLT group failed to effectively improve the pain pressure threshold.

## 4. Discussion

In the present study, 33 patients with two-sided knee OA were randomized into the ALT group (16 subjects) and the PLT group (17 subjects). In the course of the experiment, one subject of the treatment group withdrew after one week treatment, and two of the control group also quitted after two weeks treatment. The patients refused to continue because they felt unsure of the painless treatment and felt no effect at all. Thus, the two groups just had 15 subjects each upon completion of the four-week treatment course. These patients were with two-sided knee OA pain. However, neither traditional physiotherapy nor drug-assisted treatment produced good curative effect. After LLLT for three times per week for four weeks, the curative effect for the ALT group was better than that of the PLT group in terms of knee OA severity index, conscious pain index for moving knee, conscious pain index for knee resting still, and pes anserinus tendon PPT with statistically significant differences.

Degenerative arthritis initially affects the medial articular surface, so degenerated articular cartilage often occurs in the medial tibia and/or on the articular surface of the femur [26]. For patients with knee OA, these acupoints, SP9 and SP10, which anatomically correspond to vastus medialis oblique muscle and pes anserinus tendon, respectively, are the most susceptible to soreness. SP9 and SP10 are found at the points on the spleen channel. According to the acupuncture principle of “tracking and treating a disease along the appropriate meridian course,” stimulating points of the spleen channel in the vicinity of the knee joint produces curative effect on degenerative arthritis. EX-LE2 (hèdǐngxué) is the most popular extraordinary point used for managing the knee problem in traditional Chinese medicine. The acupoint is located in the center of the upper border of the patella and needled with the patient's knee slightly flexed. In the neuroanatomy of western medicine, the point is innervated via the intermediate femoral cutaneous nerve of the thigh (L2-L3).

Pain of knee OA involves arthrentasis, articular cartilage impairment, peripheral soft tissue damages, etc. LLLT can facilitate tissue repair and proliferation by promoting blood circulation, thereby relieving joint pain and improving pain pressure threshold [27–29]. Hegedus et al. [27] indicated that degenerative arthritis patients could be treated with the LLLT apparatus, twice per week for four weeks. Results showed that the treatment group showed significant improvements in terms of pain index, pressure sensitivity, and joint bending angle after LLLT. In addition, lesions were detected using the infrared apparatus and the temperature at the targeted spots of knee joints slightly increased, thus indicating that LLLT can improve the blood circulation of the irradiated spots.

Laser penetration in human tissues varies with wavelength; that is, red laser penetrates more deeply than violet light, green light, or yellow light. Infrared ray with longer wavelength is invisible and it penetrates human tissues more deeply than visible red light. The laser apparatuses used in the present study have bichromatic laser needles alternating, having broader penetration range. Laser penetration often varies with lesion depth, and the broader penetration range can produce a better curative effect. The first bichromatic laser apparatus (685 nm/785 nm) was developed by the University of Paderborn, Germany, and the earliest related scientific research was conducted and published by Medical University of Graz, Austria, in 2002. Bichromatic laser is often a combination of two different wavelengths. For instance, the dual-frequency LLLT apparatus used in the present study combines red light (780 nm) and near-infrared light (830 nm), having deeper penetration (2-3 cm) than any laser of a single wavelength in human tissues [30].

The findings of this study have to be seen in light of two limitations. First, the sample size of this study is small and it may affect statistical power. The second limitation concerns the lack of long-term follow-up for evaluation of clinical efficacy after LLLT. Therefore, if there are sufficient resources, future research is required to follow up longer for better understanding of the subsequent responses.

## 5. Conclusions

In this study, patients with knee OA received LLLT at the three points, namely, SP9, SP10, and EX-LE2. Results showed that a diode laser (TL-816-2, Transverse Inc., Taiwan) produced a dual-frequency laser beams (combines red light (780 nm) and near-infrared light (830 nm)) and a good curative effect on knee OA, with significant improvements in the severity index (Lequesne index), conscious pain index, and pain pressure threshold. Nonetheless, the clinical efficacy of LLLT and the parameter setting of the laser apparatus need further investigation.

## Figures and Tables

**Table 1:** Baseline characteristics and clinical evaluation indicators of the subjects in the two groups.

		ALT group	PLT group	*P* value
Age (year)		70.53 ± 6.89	69.73 ± 6.91	*P* > 0.05
BMI (kg/m^2^)		26.61 ± 4.33	25.98 ± 2.71	*P* > 0.05
Duration of knee pain (month)		136.40 ± 84.08	129.20 ± 57.23	*P* > 0.05
Sex	Male (number)	1	2	*P* > 0.05
	Female (number)	14	13	*P* > 0.05
K-L grade	2 (number)	11	14	*P* > 0.05
	3 (number)	16	15	*P* > 0.05
	4 (number)	3	1	*P* > 0.05

Lequesne index		11.93 ± 4.55	12.23 ± 4.14	*P* > 0.05
Conscious VAS for moving knee		6.67 ± 2.16	6.93 ± 1.79	*P* > 0.05
Conscious VAS for resting knee		3.00 ± 1.93	3.33 ± 1.68	*P* > 0.05
Pes anserinus tendon PPT (Kg)		11.71 ± 1.86	11.81 ± 1.89	*P* > 0.05

Data are expressed as mean ± SD; ALT, active laser treatment; PLT, placebo laser treatment; BMI, body mass index; K-L grade, Kellgren—Lawrence radiographic grade of knee OA; VAS, visual analog scale; PPT, pain pressure threshold.

## Data Availability

The original data used to support the findings of this study are available from the corresponding author upon request.
